# Seasonality of SARS-CoV-2 Infections in G20 Countries: Ecological Analysis of Publicly Available Data

**DOI:** 10.21315/mjms2022.29.5.16

**Published:** 2022-10-28

**Authors:** Munetaka Ueda, Makoto Yoshida, Erika Yamashita, Divya Bhandari, Michioki Endo, Akihiko Ozaki

**Affiliations:** 1 Orthopedics Surgery, University of Tokyo, Tokyo, Japan; 2 Faculty of Medicine, Teikyo University, Tokyo, Japan; 3 Medical Governance Research Institute, Tokyo, Japan; 4 Breast Surgery, Jyoban Hospital of Tokiwa Foundation, Fukushima, Japan

**Keywords:** SARS-CoV-2, seasons, quaratine, epidemiologic research design, public policy

## Abstract

Previous laboratory studies of severe acute respiratory syndrome coronavirus 2 (SARS-CoV-2) revealed that the stability of the virus in the air or on surfaces is sensitive to seasonally relevant environmental conditions. However, the seasonality of the virus in the real world remains unclear because each country adopted various infection control policies. Therefore, we investigated peak dates with regard to new confirmed cases of coronavirus disease 2019 (COVID-19) and an association of these dates with the timing of the lockdown among G20 countries that have four seasons from 1 June 2020 to 18 February 2021. As a result, countries in both hemispheres experienced seasonal peaks in the number of COVID-19 cases both in the middle of warm and cold seasons. In addition, there were no apparent relationships between the peak date and periods with stringent measures. Our study demonstrated that SARS-CoV-2 causes seasonal outbreaks in the winter and possibly summer and thus, countries might need to consider measures to prepare for resurgence of the virus in the middle of 2021.

## Introduction

Despite the worldwide implementation of various intervention measures to control the coronavirus disease 2019 (COVID-19) pandemic, which is caused by severe acute respiratory syndrome coronavirus 2 (SARS-CoV-2), many countries in the northern hemisphere experienced a resurgence of infections during the winter season of 2020/2021 (1). Laboratory studies on SARS-CoV-2 revealed that the stability of the virus in the air or on surfaces is sensitive to seasonally relevant environmental conditions, such as humidity, temperature and sunlight (2, 3). In addition, other coronaviruses are generally known to be prevalent in the winter (4). A better understanding of seasonality can provide insights on when and where the sudden surge of infection is likely to occur and can be beneficial in formulating public health interventions and preventing its transmission to some extent.

In this context, a recent ecological study of 10 countries suggested that the infectivity and mortality of SARS-CoV-2 would be higher during cold seasons (5). However, this study did not take into account the effect of infection control measures in each country on the transmissibility of SARS-CoV-2. Therefore, we investigated peak dates with regard to newly confirmed cases of COVID-19 and an association of these dates with the timing of lockdowns among countries in the Group of Twenty (G20) that have four seasons.

## Methods

The countries included in the study were the G20 countries located in the temperate or subarctic zones according to the Köppen climate classification.

We obtained data on the daily number of newly confirmed cases from the ‘Our World in Data’ website from 1 June 2020 to 18 February 2021 (6). The 1-week, 2-week and 4-week averages of newly infected persons were used to identify a single ‘infection wave’ (see Supplementary Material). The ‘peak date’ was defined as the date on which the 1-week average of new infections was the largest during the ‘infection wave.’

We also collected data from various news websites on the period when the most stringent infection control measures (lockdowns or other comparable policies) were implemented.

## Results

**Figure 1:**
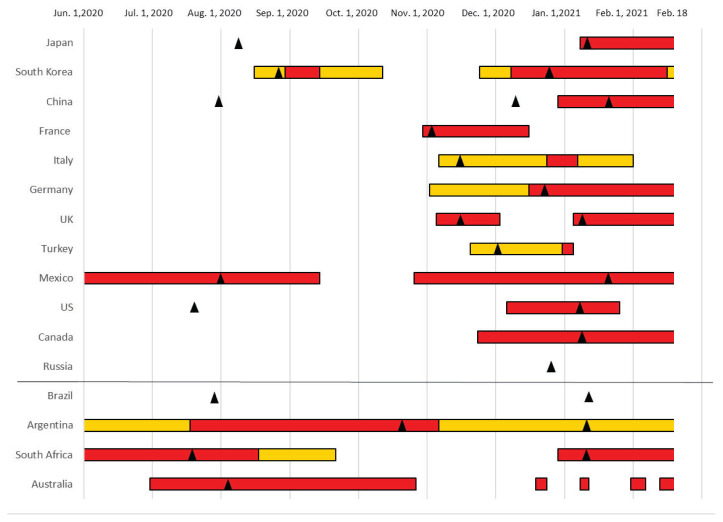
Periods with the most stringent infection control measures and peak dates Notes: red lines = periods with the most stringent measures; yellow lines = periods with stringent measures, though not the most stringent; black triangles = peak dates

As shown in Figure 1, there were seasonal peaks in the number of COVID-19 cases during both warm and cold seasons. In the northern hemisphere, all of the included countries experienced a peak between November 2020 and January 2021; some countries also experienced a peak between July and August 2020. In the southern hemisphere, most countries experienced a peak between July and August 2020 and in January 2021.

In addition, there were no apparent relationships found via a visual inspection between the timing of the peak in the confirmed cases and periods with stringent measures. Concretely, the interval between the beginning of stringent policies and the peak date from November 2020 to January 2021 varied greatly among the studied countries (3 days–224 days). Furthermore, there were no apparent differences in the peak dates between the countries with and without stringent measures.

## Discussion

Our study showed the surge of new COVID-19 cases during the cold season, which is consistent with the results of the previous ecological study in 10 countries (5). Our findings also align with the seasonality of other viral respiratory infections, such as human coronaviruses, influenza viruses and respiratory syncytial virus, which tend to peak during the winter season (7).

Moreover, our study showed that SARS-CoV-2 may be prevalent during the warm season, which is also observed with other coronaviruses. Although most coronaviruses are dominant during the winter season, observational studies in Hong Kong and Israel revealed that coronavirus HKU1 and human coronavirus NL63 are prevalent during the summer season (8, 9). Additionally, in a systematic review of 22 cohort studies set in the northern hemisphere, the proportion of studies that have reported more than 5% of specimens that were tested positive for human coronavirus exceeded 15% from June to August, which was higher than in May, September and October (4).

## Conclusion

Our findings should be interpreted considering the following limitations. Our study did not consider other confounding factors (e.g. non-pharmaceutical interventions and new virus variants) that could have distorted the findings to some extent. The duration of our study period was also limited.

Nonetheless, our study provides insight into the seasonality of the SARS-CoV-2 virus. Our findings highlight the need to consider measures to prepare for the resurgence of the virus in the middle of 2021. More detailed and comprehensive studies are needed to fully understand the seasonality of this virus.

## Supplementary Material

We obtained data on the daily number of newly confirmed cases from the ‘Our World in Data’ website from 1 June 2020 to 18 February 2021 (1). For each day, we calculated the average number of new infections over the past 1 week, 2 weeks and 4 weeks (the respective values are shown as average_1w[date], average_2w[date] and average_4w[date]). We used these moving average values to minimise the day-of-the-week effect on the number of tests.

For each day, if all four of the following conditions were met, the day was considered one in which there was a trend toward the spread of infection (we called this the ‘increasing day’).

1. average_1w(date)>average_1w(previous day)
2. average_2w(date)>average_2w(previous day)
3. average_1w(date)>average_2w(date)
4. average_2w(date)>average_4w(date)

Similarly, for each day, if all of the following conditions were met, the day was determined to be one in which there was a trend toward the containment of infection (we called this the ‘decreasing day’).

1. average_1w(date)<average_1w(previous day)
2. average_2w(date)<average_2w(previous day)
3. average_1w(date)<average_2w(date)
4. average_2w(date)<average_4w(date)

Days when neither the conditions for ‘increasing days’ nor ‘decreasing days’ were met were defined as ‘inconclusive days.’

If ‘increasing days’ occurred consecutively within 1 week, or if the two ‘increasing days’ were more than 2 weeks apart with ‘increasing days’ or ‘inconclusive days’ in between but without ‘decreasing days,’ the period was judged to be the ‘expansion stage.’ Similarly, if ‘decreasing days’ occurred consecutively within 1 week or if the two ‘decreasing days’ were more than 2 weeks apart, with ‘decreasing days’ or ‘inconclusive days’ in between but without ‘increasing days,’ the period was judged to be the ‘contraction stage.’

The period from the first day of the ‘expansion stage’ to the last day of the ‘contraction stage’ was defined as an ‘infection wave.’ The ‘peak date’ was defined as the date on which the average_1w(date) was the largest during the ‘infection wave.’

Reference1RoserMRitchieHOrtiz-OspinaEHasellJCoronavirus pandemic (COVID-19) [Internet]2020(Retrieved 2021 Feb 20). Available at: https://ourworldindata.org/coronavirus
